# One-year patency of a novel biorestorative polymeric coronary artery bypass conduit

**DOI:** 10.1093/icvts/ivaf006

**Published:** 2025-01-11

**Authors:** Isaac George, Paulo Neves, Martijn Cox, Adrian Ebner

**Affiliations:** Division of Cardiothoracic Surgery/Structural Heart & Valve Center, New-York Presbyterian Hospital, Columbia University Medical Center, New York, NY, USA; Xeltis BV, Eindhoven, Netherlands; Xeltis BV, Eindhoven, Netherlands; Cardiac Surgery Department, Sanatorio Italiano, Asunción, Paraguay

**Keywords:** CABG, coronary bypass, saphenous vein, cardiac surgery

## Abstract

Coronary artery bypass graft (CABG) surgery remains the gold standard in the treatment of complex coronary artery disease. Saphenous vein grafts (SVG) are commonly used for the non-left anterior descending artery. However, SVG failure rates in CABG surgery have been reported to be as high as 30% at 1 year and ∼50% at 10 years. Despite suboptimal performance, ∼80% of all CABG surgery includes SVG graft use. Therefore, an off-the-shelf, small-diameter vascular conduit with good patency rates remains a large unmet clinical need. XABG (Xeltis BV, Eindhoven, The Netherlands), a novel supramolecular electrospun biorestorative polymeric conduit with an embedded nitinol microskeleton, is under clinical development to fulfill this unmet need. This case report aims to demonstrate the safety and feasibility of this conduit in a routine CABG operation with implantation in a 70-year-old male patient with three-vessel disease. The XABG conduit remained patent with TIMI 3 flow at 6 months, and patency was confirmed by cardiac computed tomography at 12 months.

## INTRODUCTION

Coronary artery bypass grafting (CABG) remains an optimal therapy for coronary artery disease (CAD), particularly in patients with complex multivessel disease. However, autologous saphenous veins are not without limitations. Reported saphenous vein graft (SVG) failure rates in CABG surgery are 10–30% at 1 year and 50% at 10 years [1].

**Figure 1:**
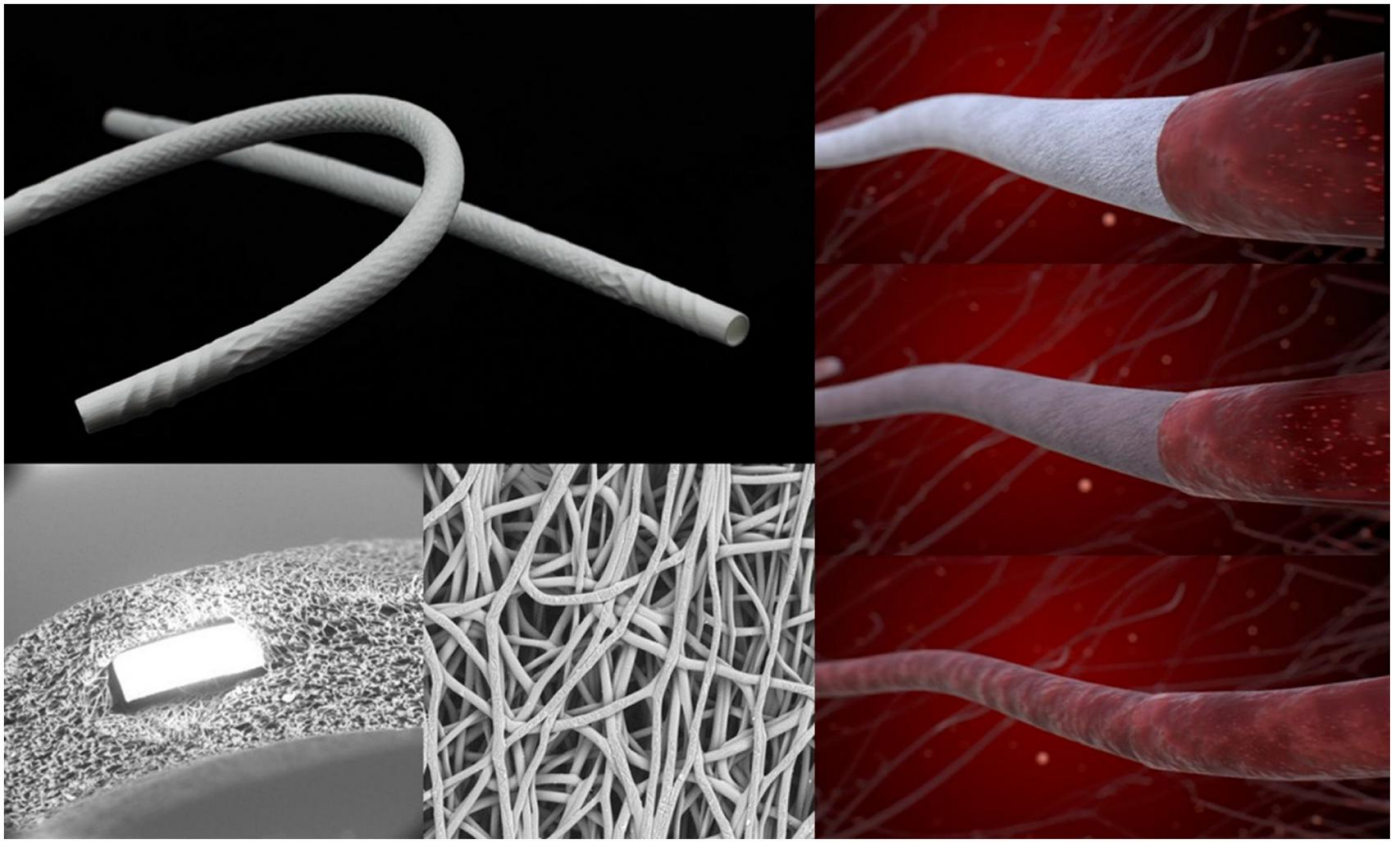
XABG conduit. A representative photograph of the XABG conduit is presented in the left upper panel. A representative scanning electron photomicrograph of the outer surface and cross-section of a device is shown in the lower left. Right panel provides a graphical representation of the remodelling process

Therefore, an off-the-shelf, small-diameter vascular graft that maintains long-term patency in CABG could revolutionize treatment options for millions of patients each year. XABG (Xeltis, Eindhoven, The Netherlands) is a novel supramolecular electrospun biorestorative polymeric conduit [2]. It is 4 mm in diameter, 500 µm in wall thickness and 12 cm in length (Fig. 1). The conduit is porous and designed to allow instant blood cells permeation. Immediately after implantation, the device acts as a functional conduit and, over time, provides a mechanical and structural scaffold for cell ingrowth propelled by the body’s natural healing process.

**Figure 2:**
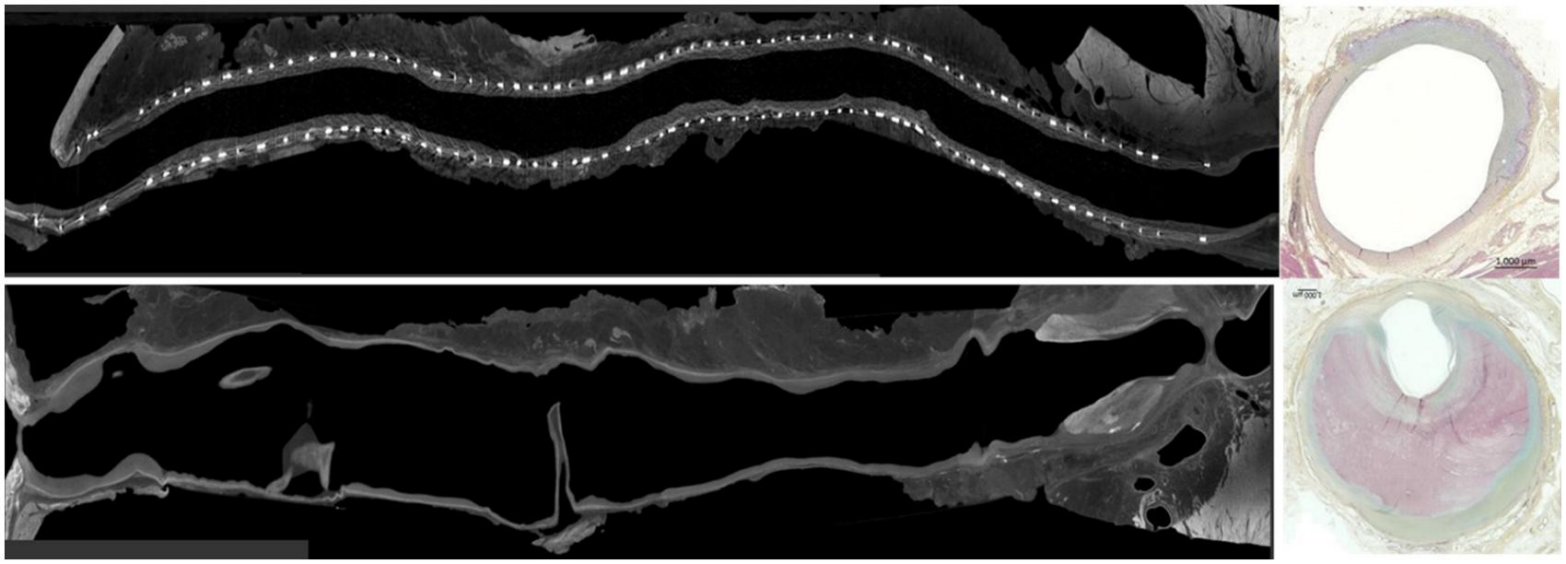
Micro CT profiles of 12-month ovine explants comparing XABG (upper left) and saphenous vein graft (SVG) control (lower left). Both were implanted for 12 months in an ovine CABG model. Right: histology cross sections near distal anastomosis of each graft (upper right, XABG; lower right, SVG). Figure adapted from Ono *et al*.[3]. CT: computed tomography

XABG underwent extensive preclinical testing, which included bench testing following a rigorous good laboratory practice animal study, which showed excellent patency at 12 months in a challenging ovine CABG model—descending aorta to LAD. In this study, 6 months XABG patency was 93%, and 12-month patency was 73%. When compared with a SVG control group, XABG showed less distal anastomosis stenosis, improved flow and less dilatation. Histology analysis demonstrated less neointima hyperplasia in the XABG group when compared with SVG (Fig. 2) [3]. The XABG conduit is under clinical trials and is not approved for commercial use in the USA or in the EU.

## METHODS

### Ethical statement

Ethical approval for this study was obtained from the Paraguayan Institute of Social Studies Ethical Committee (reference number CIE/IPES 048/23). Written consent was obtained for this patient.

The patient, a 70-year-old male with a history of severe three-vessel CAD, was selected for XABG use due to the unavailability of suitable autologous grafts, namely SVG. Preoperative assessment included coronary angiography, which revealed significant stenosis in the left anterior descending (LAD) artery and right coronary artery (RCA) as assessed by Quantitative Flow Ratio (QFr, Medis Suite, Medis Medical Imaging, Leiden, The Netherlands). LAD proximal lesion QFr was 0.66, LCx proximal lesion QFr was 0.92 and the proximal lesion RCA QFr was 0.76. The RCA showed good diameter (2.2 mm) in the target area for the anastomosis, which, in combination with a severe proximal lesion, minimizes the risk of occlusion. Therefore, the surgical plan was to graft the LAD and RCA. The decision was made to utilize the XABG graft to the RCA.

## RESULTS

The operation was conducted in a standard fashion. After sternotomy, cardiopulmonary bypass (CPB) was initiated via ascending aorta and right atrial appendage. An aortic cross-clamp was applied, and blood cardioplegia delivered with an initial dose and subsequently antegrade every 20 min. After application of an external sealant and subsequent hydration (total of 10 min preparation time), the XABG conduit was implanted, with the distal anastomosis done first, using a prolene 7–0 running suture. After the LIMA to LAD anastomosis, the proximal XABG conduit anastomosis was done with prolene 6–0 running suture. CPB weaning was uneventful. Prior to sternotomy closure, selective XABG conduit catheterization using a 5 Fr JR4 catheter via the right femoral artery was performed and XABG graft patency was confirmed (Video 1).

The postoperative course was uneventful, and the patient was discharged on the post-operative Day 7 under dual antiplatelet therapy with aspirin 150 mg id and ticagrelor 90 mg bid, which was started on postoperative day 1 and continuously maintained until 12 months. One- and 6-month postoperative angiography demonstrated a patent conduit with TIMI 3 flow (Video 1). Twelve months postoperative cardiac computed tomography showed a patent conduit with no lumen irregularities or stenoses (Video 1). The patient remained asymptomatic during this period.

## DISCUSSION

The use of a polymeric conduit in CABG represents a significant advancement in coronary revascularization. This novel graft material addresses key limitations of traditional autologous grafts, such as lack of availability, donor site morbidity and variable patency rates.

In this case report, the XABG conduit showed excellent intraoperative handling and postoperative outcomes, with no complications and sustained patency at 12 months. These results suggest that XABG may provide a reliable alternative to SVG, particularly in patients lacking suitable autologous grafts.

Further research is necessary to validate these findings in larger, diverse patient populations and over longer follow-up periods. The potential for XABG to improve long-term outcomes and reduce the need for reinterventions could significantly impact patient care and healthcare costs.

## Data Availability

The data underlying this article will be shared on reasonable request to the corresponding author.
